# Complete Annotated Genome Sequences of Two Shiga Toxin-Producing *Escherichia coli* Strains and One Atypical Enteropathogenic *E. coli* Strain, Isolated from Naturally Colonized Cattle of German Origin

**DOI:** 10.1128/genomeA.00321-17

**Published:** 2017-05-11

**Authors:** Lutz Geue, Christian Menge, Christian Berens, Stefanie A. Barth

**Affiliations:** Friedrich-Loeffler-Institut/Federal Research Institute for Animal Health, Institute of Molecular Pathogenesis, Jena, Germany

## Abstract

Shiga toxin-producing *Escherichia coli* (STEC) strains are important zoonotic enteric pathogens with the main reservoir in cattle. Here, we present the genomes of two STEC strains and one atypical enteropathogenic *E. coli* strain from cattle origin, obtained during a longitudinal study in German cattle herds.

## GENOME ANNOUNCEMENT

Shiga toxin-producing *Escherichia coli* (STEC) strains comprise a group of zoonotic enteric pathogens (1). The main reservoirs for STEC strains are ruminants, with cattle in particular. In humans, STEC infection may result in diarrhea, frequently complicated by the onset of hemorrhagic colitis or several renal and neurological sequelae, including hemolytic uremic syndrome (2–5). Atypical enteropathogenic *E. coli* (aEPEC) (6), lacking both *stx* bacteriophages as well as *bfpA*, were also detected in cattle herds. We report here the genome sequences of two persistent STEC strains with different serotypes, O182:H25 (strain 13E0725) and O156:H25 (strain 13E0780), but an identical sequence type, ST300. Additionally, we sequenced a sporadic aEPEC strain of serotype O156:H8 (strain 13E0767) and sequence type ST327. All strains were isolated during a longitudinal study investigating the prevalence of STEC in cattle (7).

Genomic DNA was extracted from overnight cultures in Luria-Bertani broth using the ZR fungal/bacterial DNA kit (Zymo Research Europe GmbH, Germany). The whole-genome sequences were acquired by a commercial service provider (GATC, Konstanz, Germany) using a PacBio RSII system (Pacific Biosciences, USA) with single-molecule real-time technology. Subsequent *de novo* assembly following the HGAP3 protocol yielded a single polished contig with 200-fold average reference coverage. In order to ensure closed-circle conformation of the bacterial chromosome, mapping, sequence analyses, and annotation were carried out using the commercial software package Geneious version 9.1.6 (Biomatters Ltd., New Zealand).

The strains 13E0725, 13E0780, and 13E0767 have circular complete genomes of 5,112,484 bp, 5,372,291 bp, and 4,942,246 bp, respectively. The genomes were annotated by NCBI’s Prokaryotic Genomes Annotation Pipeline.

### Accession number(s).

These whole genomes have been deposited at DDBJ/EMBL/GenBank under the accession numbers CP020092 (13E0725), CP020106 (13E0780), and CP020107 (13E0767). The versions described in this paper are the first versions, CP020092.1, CP020106.1, and CP020107.1.
